# An investigation into the present status and influencing factors of nurse retention in grade-a tertiary general hospitals in Shanxi Province within the framework of the magnet hospital concept

**DOI:** 10.1186/s12913-024-10945-w

**Published:** 2024-04-10

**Authors:** Li-Hong Yue, Lin-Ying Wang, Jin-Li Guo, Wan-Ling Li, Jian-Wei Zhang

**Affiliations:** 1 Department of Infection, Tongji Shanxi Hospital, Shanxi Bethune Hospital, Shanxi Academy of Medical Sciences, Third Hospital of Shanxi Medical University, 030032 Taiyuan, China; 2 Department of Nursing, Tongji Shanxi Hospital, Shanxi Bethune Hospital, Shanxi Academy of Medical Sciences, Third Hospital of Shanxi Medical University, No.99 of Longcheng Street, Xiaodian District, 030032 Taiyuan, China; 3 Department of Nursing, Second Hospital of Shanxi Medical University, 030001 Taiyuan, China; 4 Department of Geriatrics, Tongji Hospital, Tongji Medical College, Huazhong University of Science and Technology, No.1095 of Jiefang Avenue, Qiaokou District, 430030 Wuhan, China

**Keywords:** Grade-A tertiary general hospitals, Influencing factor, Magnet hospital, Willingness to continue working

## Abstract

**Background:**

The attrition of nursing staff significantly contributes to the shortage of healthcare professionals. This study entailed an examination of the propensity of nurses to sustain employment within Grade-A tertiary general hospitals and the various influencing factors.

**Methods:**

A total of 2,457 nurses from three grade-A tertiary general hospitals were surveyed. The survey instruments included a general information questionnaire, a scale measuring their willingness to continue working, and a Chinese version of the Magnet Hospital Factor scale.

**Results:**

The scores of the willingness to continue working scale and the Magnet Hospital Factor scale were 21.53 ± 4.52 and 145.46 ± 25.82, respectively. There were statistically significant differences in the scores of willingness of nurses to continue working across various factors, including the department, age, marital status, family location, length of service as nurses, professional title, position, and employment type, upon comparison (*P* < 0.001). The correlation analysis showed that there was a positive correlation between the willingness of nurses to continue working and the magnet hospital factors, with a correlation coefficient of 0.523 (*P* < 0.01). Regression analysis showed that department, length of service as nurses, professional title, position, average monthly income, number of night shifts, medical care relationship, educational support, and nursing manager support among the magnet hospital factors were important predictors of willingness to continue working (*P* < 0.001).

**Conclusion:**

The willingness of nurses to continue working in grade-A tertiary general hospitals in Shanxi Province was determined to be at an upper-middle level. The magnet status of grade-A tertiary general hospitals needs to be improved, and there are many factors that influenced willingness of nurses to continue working. To cultivate a more favorable environment and bolster nurse recruitment and retention, all healthcare institutions should strive to establish a magnet nursing environment, thereby fostering the robust development of the nursing team.

## Background

As of the conclusion of 2020, China’s ratio of registered nurses per 1,000 individuals stood at 3.34, a figure that remains below the World Health Organization’s 2014 recommendation of maintaining a ratio exceeding 5 registered nurses per 1,000 people. In Norway, the ratio of registered nurses per 1,000 individuals was 17.27, in Japan, it was 11.49, and in the United States, it was 9.8, as reported by Cn-healthcare in 2016 [1]. The World Health Care 2020 report indicates an anticipated nurse shortage of 5.7 million by the year 2030 [2]. The shortage of nurses has multifaceted implications, impacting not only the quality of nursing care and patient safety but also the physical and mental well-being of nurses. Nurse resignation stands out as a significant contributor to this shortage. The turnover rate among nurses in China’s tertiary hospitals ranges from 5.8 to 12%. Findings from a survey conducted by China’s Nursing Quality Control Center reveal that 17.86% of nurses express an intention to leave their current positions, with 83% of them considering leaving the nursing profession altogether. Approximately 27% of nurses who have left their positions have transitioned to non-nursing professions, and only 67.89% of nursing graduates choose to pursue a career in nursing [3], underscoring the need for robust strategies in recruiting and retaining nurses.

The willingness of nurses to continue working was initially introduced by Mowday et al. [4] in 1979. In 2001, Price standardized this concept, defining it as the inclination of nurses to remain in their roles after a comprehensive evaluation of their present work conditions and future professional development prospects [5]. Studies have confirmed that increasing the willingness to continue working can increase the retention of nurses and reduce the turnover rate [6–9]. Currently, there is a dearth of large-sample empirical studies on the willingness of nurses to continue working in Shanxi Province. Existing research on the influencing factors of this willingness predominantly revolves around demographic, psychological, and organizational factors [10–20]. The concept of a ‘magnet hospital’ was first introduced by McClure et al. in 1981 [21]. This term was coined to characterize hospitals that demonstrated proficiency in attracting and retaining nursing staff, particularly in the face of elevated nurse turnover rates. Findings from various studies suggest that magnet hospitals have the potential to enhance the practical experience of nurses and mitigate turnover rates [22]. Therefore, in this study we investigated the willingness of nurses to continue working in grade-A tertiary general hospitals, the highest tier of hospitals in China, located in Shanxi Province. We further explored the influencing factors from the perspective of magnet hospitals, with the objective of fostering nurses’ sustained commitment to their roles. We also sought to delineate the contours of a magnet nursing work environment suitable for Shanxi Province, providing a foundation for enhancing the practical experience of nurses.

## Participants and methods

### Participants

The participants in this study were nurses from three grade-A tertiary general hospitals in Shanxi Province. Inclusion criteria: ① A practicing nurse certificate from the People’s Republic of China (PRC); ② Worked in a registered medical and health institution for at least 1 year; ③ No history of severe physical and mental illness or psychological disorder; ④ Informed consent and voluntary participation in the study. Exclusion criteria: ① Under training nurses; ② Pregnant nurses; ③ Absent nurses due to personal leave, sick leave, public holiday, etc.; ④ Rehired nurses after retirement. Nurses were excluded from the study based on specific criteria, including being training nurses who sought professional knowledge and practice without the primary aim of employment, and thus experiencing lower work and psychological pressures than unit employees. Additionally, pregnant nurses, given the physical demands of their work, were excluded due to increased fatigue, which could elevate the likelihood of considering leaving their jobs. Throughout the survey period, the occurrence of factors such as accidental leave, sick leave, and sabbatical leave resulted in the absence of nurses, potentially impacting their responses to the survey questionnaires. The study underwent review by the Hospital Ethics Committee.

### Methods

#### Investigation tool

The investigation was carried out through the administration of an electronic questionnaire. The data collection occurred at Shanxi Baiqun Hospital from January 24th to 28th, 2022, at the People’s Hospital of Xinzhou City from February 21st to 22nd, 2022, and at the Second Hospital of Shanxi Medical University from May 17th to May 21st, 2022. The survey design drew significant inspiration from previous studies. In a departmental meeting, the head of the Nursing Department outlined the study’s objectives, content, and provided instructions for completing the questionnaire. The survey QR code was distributed, and participants were informed of the questionnaire deadline. Ensuring anonymity was a paramount consideration in this survey, achieved by omitting any fields for participant names in the questionnaire. ① General information questionnaire: Mainly included age, gender, marital status, educational background, professional title, and working life. ② The scale measuring the willingness of nurses to continue working was translated and revised by Tao and Wang [23]. Comprising 6 items, the scale utilized Likert’s 5-level scale, incorporating questions such as contemplating leaving the current job, frequency of job-seeking activities, and willingness to quit nursing. A reverse scoring system was applied, and the total score for the scale was calculated as the sum of individual item scores. A higher total score indicated a greater inclination to continue working. The Cronbach’s α coefficient for the scale ranges from 0.742 to 0.759, indicating a high level of reliability and validity [20]. ③ Chinese version of the magnet factor scale: The scale utilized in this study was initially developed by American scholar Kramer and subsequently revised for localization by Pan et al. [24, 25] It encompasses 7 dimensions and comprises 45 items. Employing Likert’s 4-level scale, ranging from “strongly disagree” to “strongly agree,” with corresponding scores of 1–4, the total score for the scale falls within the range of 45–180. A higher score indicates a greater level of the hospital’s magnet factor. The validity of the scale ranges between 0.743 and 0.906, and its internal consistency reliability is reported to be between 0.831 and 0.972.

#### Investigation methods

For this study, the convenience sampling method was employed to select Shanxi Bethune Hospital, the Second Hospital of Shanxi Medical University, and Xinzhou People’s Hospital. The research group initiated contact with the head of the nursing department at each selected hospital. Following a detailed explanation of the research purpose, content, and questionnaire filling method using standardized instructions, a questionnaire link was sent to the participants through the WJX platform. The designated individual in charge of each hospital disseminated the questionnaire link among the nursing staff, clarifying that the link to the informed consent form could be accessed on the questionnaire homepage. To commence the questionnaire, participants were required to click on the “Informed Consent” section. Throughout the survey, submission of completed questionnaires was limited to a single instance from each unique IP address. All collected information remained confidential, visible solely to the data analyst. A total of 2,464 questionnaires were collected, and following the exclusion of questionnaires demonstrating apparent patterns or logical inconsistencies in responses, 2,457 valid questionnaires were retained. This resulted in an effective recovery rate of 99.72%.

#### Statistical methods

The data obtained from the WJX platform, were analyzed using SPSS 22.0 statistical software. Measurement data are presented as mean ± standard deviation, and *t*-tests were employed. Count data are expressed as percentages and analyzed using the chi-squared test. Pearson’s correlation analysis, one-way analysis of variance (ANOVA), and binary logistic regression analysis were utilized. A significance level of *P* < 0.05 was deemed statistically significant.

## Results

### General information

There were 103 males and 2,354 females among the 2,457 nurses; ages ranged from 21 to 30 (33.18 ± 6.83) years. In terms of work experience, 123 nurses had worked for ≤ 1 year, 539 nurses had worked for 1–5 years, 841 nurses had worked for 6–10 years, 542 nurses had worked for 11–15 years, 167 nurses had worked for 16–20 years, 83 nurses had worked for 21–25 years, and 162 nurses had worked for ≥ 26 years; There were 1,845 contract nurses and 612 nurses on regular payroll. There were 552 nurses who had no night shift every month, 678 nurses had 1–5 night shifts, 1,075 nurses had 6–10 night shifts, and 152 nurses who had 11 night shifts or more.

### Willingness of nurses to continue working and magnet hospital factors score

The total score on the willingness of nurses to continue working scale was 21.53 ± 4.52, with an average item score of 3.59 ± 0.75. The highest individual item score was observed for the question “How often do you seek a new job,” with a score of 4.43 ± 0.82. The detailed scores for each item are presented in Table 1.

**Table 1:** Nurses’ willingness to continue working scale score

Items	Item scores	Sequence
1. The likelihood of you continuing to engage in nursing work	4.15 ± 0.88	2
2. Would you consider leaving your current nursing job if you had other job opportunities	2.59 ± 1.13	6
3. How often do you search for a new job (e.g., reading information in a newspaper or advertisements, making phone calls, sending out resumes, etc.)	4.43 ± 0.82	1
4. You have never considered leaving your nursing job	3.08 ± 1.26	5
5. You are not looking for a new job (non-nursing) next year	3.87 ± 1.10	3
6. Which of the following statements clearly reflects your thoughts	3.42 ± 0.98	4

Regarding the magnet hospital factor scale, the total score was 145.46 ± 25.82, and the average score for each item was 3.23 ± 0.11, corresponding to a score rate of 80.81%. Among the dimensions, “cultural values” had the highest score rate at 83.40%, while “nursing practice management” had the lowest score rate at 76.44%. The scores for each dimension are outlined in Table 2.

**Table 2:** Score of each dimension in the Chinese version of the Magnet hospital factor scale (‾x ± s)

Items	Dimension scores	Average scores	Scoring rate (%)	Total dimension score
Doctor-nurse relationship	12.63 ± 2.71	3.16 ± 0.10	78.95	16
Educational support	12.70 ± 2.79	3.17 ± 0.06	79.36	16
Nursing autonomy	22.82 ± 3.91	3.26 ± 0.08	81.49	28
Nursing practice management	12.23 ± 2.82	3.06 ± 0.10	76.44	16
Nursing management support	42.39 ± 8.42	3.26 ± 0.03	81.53	52
Rational allocation of human resources	9.32 ± 2.15	3.11 ± 0.09	77.70	12
Cultural values	33.36 ± 5.75	3.34 ± 0.05	83.40	40

Note: Scoring rate = Actual score/Total dimension score ×100

### Factors affecting willingness of nurses to continue working

#### Comparison scores of willingness of nurses to continue working with different demographic characteristics

Significant differences were found in the scores of willingness of nurses to continue working with respect to departments, age, marital status, family location, service experience as nurses, professional title, position, employment method, average monthly income, and the number of night shifts per month (Table 3).

**Table 3:** Comparison of nurses’ willingness to continue working scores with different demographic and working characteristics (‾x ± s)

Items	N	Score of willingness to continue working	Statistical values	*P*-value
Department			F = 7.172	0.000
Internal medicine	820	21.12 ± 4.593		
Surgery department	853	21.64 ± 4.323		
Department of gynecology and obstetrics	164	22.37 ± 4.458		
Pediatric department	74	19.74 ± 4.913		
Oncology department	80	22.61 ± 5.01		
Emergency department	77	21.45 ± 3.999		
Intensive care unit	162	20.61 ± 4.912		
Operating room	101	23.09 ± 4.04		
Others	126	22.76 ± 4.148		
Age			F = 35.901	0.000
≤ 25 years old	271	20.73 ± 4.197		
26–35 years	1497	21.11 ± 4.528		
36–45 years	513	22.2 ± 4.322		
≥ 46 years	176	24.4 ± 4.232		
Gender			t=—1.554	0.120
Male	103	20.85 ± 4.351		
Female	2354	21.56 ± 4.529		
Educational qualification			F = 1.692	0.184
Undergraduate	86	22.4 ± 4.444		
Graduate	2322	21.5 ± 4.525		
Post-graduate	49	21.73 ± 4.466		
Marital status			t=-4.495	0.000
Unmarried	653	20.85 ± 4.398		
Married	1804	21.78 ± 4.543		
Location			t = 3.741	0.000
City	1803	21.73 ± 4.591		
Village	654	20.98 ± 4.283		
Years of service as a nurse			F = 18.810	0.000
≤ 1 year	123	20.68 ± 4.045		
1–5 years	539	20.71 ± 4.46		
6–10 years	841	21.24 ± 4.514		
11–15 years	542	21.69 ± 4.449		
16–20 years	167	22.32 ± 4.406		
21–25 years	83	22.71 ± 4.244		
≥ 26 years	162	24.52 ± 4.172		
Professional title			F = 30.980	0.000
Nurse	425	20.99 ± 4.187		
Senior nurse	921	21.15 ± 4.61		
Nurse-in-charge	958	21.68 ± 4.478		
Deputy director and chief senior nurse	153	24.44 ± 4.049		
Position			F = 55.320	0.000
General nurse	2118	21.35 ± 4.501		
Teaching teacher	227	21.49 ± 4.368		
Head nurse or nursing director	112	25.14 ± 3.676		
Employment method			t=-6.653	0.000
Contractual employee	1845	21.19 ± 4.494		
Regular employee	612	22.58 ± 4.45		
Average monthly income			F = 14.043	0.000
≤ RMB 3000	274	20.89 ± 4.813		
RMB 3001–6000	893	21.34 ± 4.422		
RMB 6001–9000	1032	21.53 ± 4.548		
RMB 9001–12,000	212	22.41 ± 4.152		
≥ RMB 12,001	46	25.04 ± 3.73		
Number of monthly night shifts			F = 53.787	0.000
No night shift	552	23.17 ± 4.115		
1–5	678	22.1 ± 4.517		
6–10	1075	20.41 ± 4.458		
≥ 11	152	20.99 ± 4.188		

#### Correlation analysis between the score of willingness of nurses to continue working and the score of magnet hospital factors

Pearson’s correlation analysis was performed between scores of the willingness to continue working and the scores of the magnet hospital factors. The results showed a positive correlation, with the correlation coefficient *r* = 0.523, *P* < 0.01. There was a positive correlation between the dimensions of the magnet hospital factors and the willingness to continue working, as shown in Table 4.

**Table 4:** Correlation analysis between nurses’ willingness to continue working and Magnet hospital factor scores (r)

Items	Doctor-nurse relationship	Education support	Nursing autonomy	Nursing practice management	Nursing management support	Rational allocation of human resources	Cultural values Sense of worth	Magnet hospital level
Willing to continue working in the hospital	0.463^**^	0.465^**^	0.413^**^	0.459^**^	0.498^**^	0.502^**^	0.482^**^	0.523^**^

Note: **P < 0.01

#### Binary logistic regression analysis of willingness of nurses to continue working

In the binary logistic regression analysis, the grouping of scores indicating willingness to continue working was considered the dependent variable. The variables identified as statistically significant in the single-factor analysis of general information and each dimension in the magnet hospital factor scale were utilized as independent variables. The variable assignments are delineated in Table 5. Dummy variables were established during the analysis, designating the item with assignment = 1 as the control. The finally obtained logistic model was statistically significant. The model could explain 27.9% variation in willingness of nurses to continue working. The results of multivariate analysis are shown in Table 6.

**Table 5:** Independent variable assignment

Items	Assignment method
Administrative or technical offices	Pediatric department = 1; Department of obstetrics and gynecology = 2; Emergency department = 3; Internal medicine = 4; Others = 5; Operating room = 6; Surgery department = 7; Oncology department = 8; Intensive care unit = 9
Age	≤ 25 years = 1; 26 ~ 35 years old = 2; 36 ~ 45 years old = 3; ≥ 46 years = 4
Marital status	Unmarried = 1, married = 2
Years of service as a nurse	≤ 1 year = 1; 1–5 years = 2; 6–10 years = 3; 11–15 years = 4; 16–20 years = 5; 21–25 years = 6; ≥ 26 years = 7
Professional title	Nurse = 1; Senior nurse = 2; Nurse in charge = 3; Deputy director and chief senior nurse = 4
Position	Ordinary nurse = 1; Teacher = 2; Head nurse or Nursing director = 3
Appointment method	Contract employee = 1; Regular employee = 2
Average monthly income	≤ RMB 3000 = 1; RMB 3001–6000 = 2; RMB 6001–9000 = 3; RMB 9001–12,000 = 4; ≥ RMB 12,001 = 5
Number of monthly night shifts	No night shift = 1; 1–5 = 2; 6–10 = 3; ≥ 11 = 4
Each dimension of Magnet hospital factor scale	Brought in from original value

**Table 6:** Results of multi-factor regression analysis of willingness to continue working the hospital

Independent variable	B	S.E.	Wald	Significance	OR	95% CI	
						Lower limit	Upper limit
Department			95.704	0.000			
Department (Obstetrics vs. Gynecology)	0.739	0.091	65.714	0.000	2.094	1.751	2.503
Department (Emergency vs. Pediatrics)	0.813	0.107	57.260	0.000	2.256	1.827	2.785
Department (Internal Medicine vs. Pediatrics)	0.449	0.078	33.230	0.000	1.567	1.345	1.826
Department (Others vs. Pediatrics)	0.462	0.098	22.290	0.000	1.587	1.310	1.923
Department (Operating room vs. Pediatrics)	0.639	0.103	38.751	0.000	1.895	1.550	2.318
Department (Surgery department vs. Pediatrics)	0.488	0.078	39.305	0.000	1.629	1.398	1.897
Department (Oncology vs. Pediatrics)	0.619	0.105	34.751	0.000	1.857	1.512	2.281
Department (Intensive Care Unit vs. Pediatrics)	0.418	0.092	20.864	0.000	1.519	1.270	1.818
Age			6.945	0.074			
Age (26–35 years vs. ≤ 25 years)	0.085	0.059	2.022	0.155	1.088	0.969	1.223
Age (36–45 years vs. ≤ 25 years)	0.052	0.078	0.436	0.509	1.053	0.903	1.228
Age (≥ 46 years vs. ≤ 25 years)	-0.237	0.156	2.296	0.130	0.789	0.581	1.072
Marital status (Married vs. Unmarried)	-0.047	0.043	1.208	0.272	0.954	0.876	1.038
Location (Rural vs. Urban)	0.046	0.033	1.888	0.169	1.047	0.981	1.118
Years of service as a nurse			127.395	0.000			
Years of service as a nurse (1–5 years vs. ≤ 1 year)	-0.506	0.077	43.199	0.000	0.603	0.518	0.701
Years of service as a nurse (6–10 years vs. ≤ 1 year)	-0.387	0.090	18.310	0.000	0.679	0.569	0.811
Years of service as a nurse (11–15 years vs. ≤ 1 year)	-0.207	0.098	4.407	0.036	0.813	0.671	0.986
Years of service as a nurse (16–20 years vs. ≤ 1 year)	-0.281	0.117	5.720	0.017	0.755	0.600	0.951
Years of service as a nurse (21–25 years vs. ≤ 1 year)	-0.655	0.133	24.087	0.000	0.519	0.400	0.675
Years of service as a nurse (≥ 26 years vs. ≤ 1 year)	0.205	0.186	1.218	0.270	1.227	0.853	1.766
Professional title			50.980	0.000			
Professional title (Senior nurse vs. nurse)	0.102	0.049	4.302	0.038	1.107	1.006	1.219
Professional title (Senior nurse in charge vs. Nurse)	0.073	0.059	1.555	0.212	1.076	0.959	1.207
Professional title (Deputy director and chief senior nurse vs. Nurse)	0.668	0.105	40.772	0.000	1.950	1.588	2.393
Employment method			42.699	0.000			
Employment method (Nursing director vs. Nurse)	0.128	0.047	7.546	0.006	1.137	1.037	1.245
Employment method (Head nurse vs. Nursing department director vs. Nurse)	0.593	0.096	38.103	0.000	1.810	1.499	2.185
Appointment method (Regular employee vs. Contract employee)	0.009	0.040	0.054	0.817	1.009	0.933	1.092
Average monthly income			232.456	0.000			
Monthly average income (RMB 3001–6000 vs. ≤ RMB 3000	0.381	0.047	65.354	0.000	1.464	1.335	1.606
Monthly average income (RMB 6001–9000 vs. ≤ RMB 3000)	0.635	0.050	161.969	0.000	1.888	1.712	2.082
Monthly average income (RMB 9001–12,000 vs. ≤ RMB 3000)	0.833	0.068	151.422	0.000	2.301	2.015	2.628
Monthly average income (≥ RMB 12,000 vs. ≤ RMB 3000)	1.240	0.133	86.346	0.000	3.456	2.660	4.489
Monthly night shift (s)			250.631	0.000			
Monthly night shifts (1–5 vs. no night shift)	-0.374	0.041	82.271	0.000	0.688	0.635	0.746
Monthly night shifts (6–10 vs. no night shifts)	-0.640	0.041	245.876	0.000	0.528	0.487	0.571
Monthly night shifts (≥ 11 vs. no night shifts)	-0.570	0.063	81.175	0.000	0.566	0.500	0.640
Doctor-nurse relationship	0.103	0.009	141.373	0.000	1.109	1.090	1.128
Educational support	0.085	0.010	79.752	0.000	1.089	1.069	1.109
Nursing autonomy	-0.097	0.007	181.180	0.000	0.908	0.895	0.920
Nursing practice management	0.008	0.010	0.601	0.438	1.008	0.988	1.029
Nursing management support	0.020	0.004	26.470	0.000	1.020	1.012	1.028
Rational allocation of human resources	0.207	0.014	215.514	0.000	1.230	1.197	1.265
Cultural values	0.055	0.005	101.952	0.000	1.057	1.045	1.068
Constant	-5.301	0.140	1436.045	0.000	0.005		

## Discussion

### Status of willingness of nurses to continue working in grade-a tertiary general hospitals in Shanxi Province

In this study, the willingness of nurses to continue working scale exhibited an overall score and average item score of 21.53 ± 4.52 and 3.59 ± 0.75, respectively. These scores surpassed those reported in grade-A tertiary general hospitals in Shanghai and Hangzhou [10, 26], as well as those documented in grade-A tertiary general hospitals across China by Zhang et al. [11] and tertiary traditional Chinese medicine (TCM) hospitals in China by Liu [16] Furthermore, the scores were higher than those found in male nurses in Shenzhen as investigated by Xu et al. [20] Nevertheless, the scores were lower than the reported scores of willingness of nurses to continue working during the COVID-19 epidemic investigated by Li [27] and those in military hospitals in island areas investigated by Wang et al. [17]. This suggests that the willingness of nurses to continue working in grade-A tertiary general hospitals in Shanxi Province is positioned at an upper-middle level. This observation could be attributed to the consistent advancement of nursing science and the escalating attention from the state, government, and medical institutions toward nurses. Alternatively, it may be associated with the heightened professional pride among the majority of nurses who have been at the forefront, especially during the COVID-19 epidemic. It is advisable for nursing managers to leverage both external and internal motivating factors as foundational elements, drawing insights from advanced practices both within China and internationally. Exploring affirmative strategies to foster the retention of nurses should be a priority in this endeavor.

### Magnet level of the grade-A tertiary general hospitals in Shanxi Province

In this study, the total score on the magnet factor scale for Grade-A tertiary general hospitals in the Shanxi area was 145.46 ± 25.82, with a score rate of 80.81%. This score exceeded those reported for Grade-A tertiary general hospitals in the Xinjiang area investigated by Lu et al. [28]., Grade-A tertiary general hospitals in Bengbu City examined by Lin et al. [29]., and Grade-A tertiary general hospitals explored by Liu [30], where new nurses were the research participants. Additionally, it surpassed the scores in a study conducted by Pan et al. [25] The findings indicated that the score rates for the management dimension of nursing practice and the rational allocation dimension of human resources were low. This aligns with the research outcomes reported by Lu et al. [28]. It is advisable for nursing managers to prioritize nursing practice, consolidating basic nursing care, and enhancing nurses’ proficiency in risk prediction and specialized nursing skills. Establishing open communication channels, implementing a shared governance model, and ensuring adequate human resources are essential for nursing safety. Medical institutions should strategically allocate human resources based on evaluation criteria and the specific context of Grade-A tertiary general hospitals. Promoting a magnet culture can contribute to the high-quality development of hospitals.

### Factors affecting the willingness of nurses to continue working in grade-A tertiary general hospitals in Shanxi Province

#### Effect of department on nurses’ willingness to continue working

The study results indicated that departments were significant factors influencing the willingness of nurses to continue working. Nurses in pediatrics and critical care departments exhibited lower willingness to continue working, whereas nurses in other departments demonstrated higher willingness, ranging from 1.5 to 2.2 times that of pediatric nurses (*P* < 0.001). This observation may be attributed to the nature of the work within each department and the allocation of human resources. The specific regulations governing the implementation of evaluation standards in Grade-A tertiary general hospitals in Jiangxi Province stipulate that the ratio of pediatric nurses to the actual number of open beds should not be less than 0.6:1. Similarly, the ratio of nurses in intensive medical care departments to the actual number of open beds should be no less than 2.5–3:1. Presently however, many medical institutions face challenges in meeting these requirements, and the bed-to-nursing ratio does not align with the actual clinical conditions [31], including factors such as bed utilization rates and the number of nurses engaged in non-nursing tasks. The inadequacy of human resources, high workload, responsibilities for pediatric and critically ill patients, and substantial work pressure have contributed to a diminished willingness to continue working to some extent. Hence, it is recommended that nursing managers proactively allocate nurses, tailor the working environment to the specific needs of departments, and implement incentive measures to attract and retain nursing staff.

#### Longer the working experience as a nurse, the higher the willingness of nurses to continue working

The univariate analysis results revealed significant differences in nurses’ willingness to continue working based on their work experience, indicating a gradual increase in willingness with extended service as nurses. However, the multivariate analysis indicated no significant difference in willingness to continue working between nurses with ≥ 26 years of nursing experience and those with ≤ 1 year of nursing experience. The research results were in line with those of Liu [30] and Zhuang [10]. This observation may be associated with the limited professional knowledge of junior nurses and their potential lack of a sense of belonging during rotation training. Conversely, senior nurses may have already acquired substantial subject knowledge and professional skills, along with robust interpersonal communication abilities and enhanced post competency. Research indicates that new nurses experience the greatest work pressure and highest turnover rates within their initial year of employment [32–33]. The research data also indicated that nurses with greater work experience tended to have clearer career planning [34–36], which positively influenced their willingness to continue working. It is recommended that nursing managers prioritize the professional development of junior nurses and devise personalized retention strategies tailored to their unique characteristics to enhance their willingness to continue working.

#### High professional title and high position can promote the willingness of nurses to continue working

The study results indicated that the willingness to continue working in the positions of deputy director of senior nurses and director of the nursing division was 1.95 times higher than that of a nurse (*P* < 0.001). These findings align with similar research conducted by Xu [20] on the influencing factors of willingness of male nurses to continue working in Shenzhen and by Li [27] on the influencing factors of willingness of nurses to continue working during the COVID-19 epidemic. The study revealed that the willingness of teaching nurses to continue working was 1.137 times that of common nurses, while the willingness of head nurses or nursing department directors to continue working was 1.81 times that of common nurses. These results mirror findings reported by Zhang [11] and Zhuang [10]. This phenomenon may be attributed to the fact that nurses with high professional titles or positions tend to possess specific professional and academic achievements, enjoy relatively strong work autonomy, and have increased opportunities for training and further education. It is recommended that government bodies and medical and health institutions study and promote exemplary experiences and practices. They should develop a scheme for professional title evaluation and appointment tailored to local conditions, address issues related to the professional titles of nurses, enhance the quality of professional training for junior nurses, demonstrate care for nurses, and create customized career development plans for nurses. This approach aims to facilitate nurse retention based on emotional and visionary considerations.

#### Income level is an important factor for predicting the willingness of nurses to continue working

The findings indicated that a higher income was associated with a stronger willingness to continue working, aligning with results from various studies, including those by Zhang [11] and Wei [37]. This implies that income level is a significant factor in predicting willingness of nurses to continue working. When nurses perceive a lack of proportionality between their efforts and income, they may be inclined to consider leaving their jobs, and a sustained disparity could contribute to higher turnover rates. Medical institutions and nursing managers should adopt a scientific, fair, and impartial approach in developing performance assessment programs. These programs should comprehensively consider factors such as workload, work quality, and work effects, reflecting a principle of rewarding more for higher productivity and outstanding performance. Implementing effective performance and rewards policies can contribute to a more equitable and motivating work environment for nurses.

#### The lesser the number of night shifts, the higher the willingness of nurses to continue working

The study results demonstrated a negative correlation between the number of night shifts and the willingness of nurses to continue working. Specifically, a smaller number of night shifts was associated with a higher willingness of nurses to continue working. Previous research has shown that involving nurses in the weekly shift arrangement for their department can enhance their working enthusiasm and autonomy [38]. In light of these findings, nursing managers should consider exploring department-specific night shift patterns. Scientifically determining the number and duration of night shifts and designating a day for nurses working at night as a “nurse sleep day (SD)” without additional requirements for participation in learning and training can contribute to enhancing nurses’ willingness to continue working.

#### Establishing a magnet nursing workplace can foster the willingness of nurses to continue working

The data presented in Table 4 illustrate that all dimensions of the magnet hospital factor scale exhibit a positive correlation with the willingness to continue working. The correlation coefficient between the magnet hospital level and the willingness to continue working is 0.523. The regression results further indicate that for every 1-point increase in dimensions such as healthcare relationship, education support, nursing manager support, rational allocation of human resources, and cultural values on the magnet hospital factor scale, the willingness of nurses to continue working nearly doubles. The dimension of nursing autonomy exhibited a negative correlation with the willingness to continue working, suggesting a relationship between nursing autonomy and willingness of nurses to continue working, albeit without a regression effect. This may be attributed to the presence of intermediary or confounding variables [39, 40]. Further research is needed to explore the interactions between variables and delve into potential mediating or moderating factors.

A magnet hospital is characterized as a facility that has the ability to attract high-quality nurses, akin to a magnet, and offers a practice environment that aligns with both professional and personal values [41]. The outcomes of this study suggest that the working environment in magnet hospitals positively influences the willingness of nurses to continue working, aligning with the findings of the majority of studies in this area [42–48]. Promoting equality, trust, and fostering positive collaborative relationships between doctors and nurses contributes to a favorable working atmosphere and environment for nurses. Departments, nursing departments, and hospitals should actively encourage nurses to engage in continuing education, participate in academic exchanges, and pursue further studies. Providing comprehensive support to nurses in these endeavors can significantly contribute to enhancing their professional quality and confidence. Nursing managers play a pivotal role as the “backbone” of the nursing team. Their focus should be on team building, safeguarding the interests of nurses, making informed decisions through scientific methods, ensuring optimal working conditions, and actively listening to the concerns of nurses. A judicious allocation of human resources is a fundamental prerequisite to ensure the delivery of high-quality care. Currently, in most Grade-A tertiary general hospitals, the proportion of nurses in wards and special departments falls short of established standards. This deficiency inevitably raises the workload for nurses, contributing to an elevated risk of occupational injuries, reduced job satisfaction, heightened job burnout, and an increased turnover rate among nursing staff [49]. The rational allocation of human resources should take into account the workload, considering factors such as professional titles and job levels. Utilizing flexible scheduling is essential to ensure that nurses have the necessary energy to effectively carry out clinical work. Hospital culture is a composite of widely acknowledged ideologies, value systems, and cultural forms. It encompasses the collective values, beliefs, and behavioral norms shared by the staff within the hospital [50]. An exemplary hospital culture can significantly bolster the sense of belonging and cohesion among employees, holding profound significance for the sustained development of the hospital. Medical institutions and nursing managers should prioritize the strengthening of cultural development, disseminate the values of the hospital and nursing culture, and integrate these cultural elements into the personal values of nurses.

### Research limitations and deficiencies

The study participants were drawn from Taiyuan and Xinzhou City, and it is acknowledged that there may be limitations in the representation and homogeneity of the sample. This potential lack of diversity could introduce a certain degree of bias to the research results, consequently limiting the generalizability and applicability of the findings. Furthermore, among the influencing factors considered, the analysis was limited to basic information and the impact of the magnet hospital level on the willingness of nurses to continue working. Factors such as psychological resilience, psychological pressure, and job embeddedness were not included in the analysis, and there was no in-depth exploration of how these factors might influence the willingness of nurses to continue working. This suggests a potential gap in the comprehensive understanding of the various elements influencing the willingness of nurses to continue working.

## Conclusion

Given the ongoing shortage of nurses, their willingness to continue working in Grade-A tertiary general hospitals in Shanxi Province was found to be at an upper-middle level. Various factors influenced the willingness of nurses to continue working, with the magnet hospital level showing a positive correlation with this willingness. To elevate the magnet status of these hospitals, initiatives should prioritize optimizing income structures, nurturing medical care relationships, and enhancing educational and managerial support systems. Additionally, strategic emphasis should be placed on the prudent allocation of human resources, the promotion of cultural values, and the establishment of customized magnet nursing environments. Innovative adaptations to the nursing working environment, tailored to the distinctive characteristics of the region, are recommended to effectively enhance the willingness of nurses to continue working, thereby ensuring the stability of the nursing team.

## Data Availability

All data generated or analysed during this study are included in this article. Further enquiries can be directed to the corresponding author.

## Abbreviations

PRC: People’s Republic of China
ANOVA: One-way analysis of variance
TCM: Traditional Chinese medicine
